# A Functional SNP Variant of the 3′ UTR Within the *ELF5* Gene Affects Lactation Traits Through bta-miR-487a-Mediated Regulation in Holstein Dairy Cattle

**DOI:** 10.3390/biom16081163

**Published:** 2026-08-10

**Authors:** Lingyuan Ma, Li Sun, Haotian Zhang, Chuanying Pan, Mingxun Li, Xianyong Lan, Yang Li

**Affiliations:** 1 College of Animal Science and Technology, Northwest A&F University, Yangling, Xianyang 712100, China; lingyuanma@nwafu.edu.cn (L.M.); 2024050452@nwafu.edu.cn (L.S.); zht0501@nwafu.edu.cn (H.Z.); chuanyingpan@nwafu.edu.cn (C.P.); 2 Key Laboratory of Animal Genetics & Breeding and Molecular Design of Jiangsu Province, College of Animal Science and Technology, Yangzhou University, Yangzhou 225009, China; limingxun@yzu.edu.cn

**Keywords:** Holstein cattle, lactation traits, SNP, *ELF5*, bta-miR-487a

## Abstract

The genetic and physiological variations among individuals of Holstein dairy cattle contribute to variations in milk yield. Selecting high-yield cows is critical for improving lactation performance. The present study aimed to identify functional genetic variants of the 3′ untranslated region (3′ UTR) in the *ELF5* gene associated with milk production traits and to elucidate their underlying molecular mechanisms. A total of 1314 Chinese Holstein cows were genotyped, and a novel single nucleotide polymorphism (SNP), *ELF5* g.32793 G>T, was identified in the 3′ UTR of *ELF5*. This SNP was significantly associated with milk production, such as milk yield, milk fat percentage, milk protein percentage, and somatic cell score. Bioinformatic prediction and dual-luciferase reporter assays demonstrated that the *ELF5* g.32793 G>T affects the interaction between bta-miR-487a and the *ELF5* 3′ UTR. Functional analyses in bovine mammary epithelial (MAC-T) cells showed that bta-miR-487a suppressed cell proliferation by inducing G0/G1 phase arrest and reduced the expression of proliferation-related proteins. Collectively, these findings suggested that bta-miR-487a participates in the regulation of MAC-T cell growth and that the *ELF5* g.32793 G>T polymorphism may influence lactation performance through an miRNA-associated mechanism. Therefore, the variant can be used as a functional marker for marker-assisted selection in dairy cattle breeding.

## 1. Introduction

China ranks among the world’s leading producers of dairy products. However, the lactation performance of Chinese Holstein cattle still needs improvement compared with other countries. For example, the average annual MY (milk yield) per Holstein cow in the United States was 10,603 kg, whereas in China it was approximately 8300 kg in 2020, indicating a productivity gap between the two countries’ dairy industries [1]. The Chinese dairy industry mainly relies on Holstein cows, whose lactation traits largely determine economic returns and are important indicators of milk quality [2]. Lactation traits, including MY, milk fat percentage (MFP), and milk protein percentage (MPP), are widely recognized and commonly used as primary indicators for determining both the productivity of dairy cattle and the quality of milk produced [3]. These traits are influenced by both environmental and genetic factors. Among environmental factors, management practices, heat stress, and nutrition can significantly affect lactation performance [4]. However, improvements in management and feeding have limitations, and genetic factors are likely the key to sustainably enhancing milk production. Therefore, genetic improvement of Holstein cattle represents a promising strategy to increase MY, optimize milk composition, and ultimately improve the efficiency and profitability of China’s dairy industry.

The ELF5 (E74-like ETS transcription factor 5), which belongs to the *ETS* (E26 transformation-specific) transcription factor family and is characterized as a key epithelial-specific member, was initially discovered in 1998 and has been reported to be broadly expressed in multiple tissues, including the mammary gland [5]. During the development of the mammalian mammary gland as well as the lactation process, ELF5 has been identified as a crucial regulatory factor governing mammary acinar cell lineage commitment, the morphogenesis of breast tissue, and the onset and initiation of lactation [6]. Deletion or dysregulation of ELF5 directly results in impaired acinar development and arrested mammary tissue growth, severely compromising the establishment and maintenance of normal lactation function [6]. Recent livestock research has demonstrated that ELF5 in buffalo mammary gland tissue exerts a positive regulatory effect on essential lactation-associated physiological processes, such as the proliferation of mammary epithelial cells and the synthesis of milk proteins, primarily through the modulation of classical lactation signaling pathways, including the JAK2–STAT5 axis and the PI3K/AKT1/mTOR signaling pathway [7]. Meanwhile, investigations employing an in vitro cell model such as MAC-T have shown that progesterone markedly promotes the upregulation of *ELF5* expression, along with genes associated with milk component synthesis, thereby further reinforcing the role of ELF5 as a critical functional regulator involved in milk synthesis and lactation processes in dairy cattle [8]. Although the lactation-regulatory function of *ELF5* has been preliminarily validated, current studies have mainly focused on functional characterization and signaling pathway exploration. Research on genetic variations in the *ELF5* gene in dairy cattle remains limited, particularly regarding the association between *ELF5* SNPs and lactation traits in Chinese Holstein cattle. This gap greatly restricts the application of *ELF5* in molecular marker-assisted breeding programs for dairy cattle.

MicroRNAs (miRNAs) are short regulatory non-coding RNA molecules that control gene expression after transcription by associating with complementary sequences within the 3′ UTR of mRNAs, resulting in reduced protein synthesis or enhanced transcript decay [9,10,11]. Evidences indicated that differential miRNA expression plays an important role in regulating lactation performance, mammary gland health, and reproductive traits in dairy cows [12]. For example, miR-126-3p exhibits stage-specific expression associated with MY and somatic cell score (SCS) in subsequent lactations. In addition, the ratio of miR-127 to miR-30c-5p modulates milk fat and protein synthesis, thereby influencing milk quality and yield. Furthermore, miR-142-5p, along with related miRNA combinations, is associated with key reproductive traits such as calving interval [13]. These findings suggest that miRNAs regulate multiple economically important traits through diverse mechanisms. Nevertheless, most studies have focused on single miRNA–trait associations, and it remains unclear whether miRNAs can directly target *ELF5* to modulate mammary epithelial cell proliferation and thereby influence lactation.

Therefore, this study used a total of 1314 Chinese Holstein dairy cows as the experimental population and integrated phenotypic data on lactation traits to perform association analyses for identifying SNPs in the *ELF5* gene. This approach aimed to identify functional SNPs associated with lactation traits and further elucidate the genetic mechanisms by which *ELF5* variants regulate these traits in dairy cattle. In addition, MAC-T cells were used as an in vitro model to validate miRNA-targeted regulatory effects and investigate molecular mechanisms through which miRNAs regulate mammary epithelial cell proliferation and lactation traits. This study provides new insights into the regulatory mechanisms underlying lactation traits mediated by *ELF5* and miRNAs, and further establishes a theoretical basis for molecular breeding strategies and genetic improvement in dairy cattle.

## 2. Materials and Methods

### 2.1. Blood Sample Collection

A total of 1314 Holstein dairy cows from Jiangsu Province, China, were selected for this study. All animals were raised under the same feeding and management conditions. This study followed the guidelines of the Ethics Committee of the College of Animal Science and Technology of Northwest A&F University (DK2026061566).

Blood samples were collected from the ear vein of each cow. Before sampling, the collection site was cleaned and disinfected, and blood samples were collected into anticoagulant tubes containing EDTA. The collected blood samples were immediately transported to the laboratory under cold-chain conditions and stored at −20 °C until genomic DNA extraction. Genomic DNA was extracted from blood samples and used for PCR amplification, Sanger sequencing, genotype identification, and subsequent association analysis between the identified polymorphism and milk production traits.

### 2.2. Genomic DNA Extraction

Genomic DNA was extracted using Tris-saturated phenol-phenol–chloroform methods according to the manufacturer’s protocol [14]. In brief, blood samples were lysed with lysis buffer, followed by protein digestion with proteinase K (Solarbio, P9460, Beijing, China). The lysates were extracted with an equal volume of Tris-saturated phenol and phenol–chloroform mixture to remove proteins and other impurities. After centrifugation, the aqueous phase was carefully collected, and genomic DNA was precipitated with ethanol. The DNA pellets were washed with 70% ethanol, air-dried, and dissolved in DEPC water (Solarbio, R1600, Beijing, China). The quality and concentration of extracted DNA were determined using a spectrophotometer (NanoDrop One, Thermo Fisher Scientific, Waltham, MA, USA), and DNA samples were stored at −80 °C until further analysis.

### 2.3. Cell Culture

The MAC-T and HEK-293T cell lines were kindly provided by Professor Xingping Wang (Ningxia University, Yinchuan, Ningxia, China). MAC-T and HEK-293T cell lines were grown in DMEM (HyClone, SH30243.01, Marlborough, MA, USA) containing 10% fetal bovine serum (Daxi Biotechnology, DX-1001, Hohhot, Inner Mongolia Autonomous Region, China) along with 1% penicillin–streptomycin (Solarbio, P7630, Beijing, China) and incubated at 37 °C in a humidified atmosphere of 5% CO_2_. After thawing, cells were rapidly recovered in complete medium and cultured in T25 culture flasks at 37 °C in a humidified atmosphere containing 5% CO_2_. The culture medium was replaced every day, and cells were passaged when they reached the logarithmic growth phase. Cells were expanded for several passages before being used for experiments. For cryopreservation, cells were suspended in freezing fetal bovine serum containing 10% dimethyl sulfoxide (DMSO) (Solarbio, D8371, Beijing, China) and stored in liquid nitrogen for long-term preservation.

### 2.4. Polymerase Chain Reaction (PCR)

The amplification was performed using Novagen Taq polymerase. First, a total of 9.5 μL of DEPC-treated water was dispensed into the PCR tube, after which 1 μL (100 ng) of DNA was subsequently added to the reaction mixture. Then, 1 μL of each primer was added, and finally, 12.5 μL of Taq mix was added. PCR amplification was carried out with an initial denaturation step at 95 °C for 3 min, after which 30 cycles of amplification were performed, each comprising denaturation at 95 °C for 5 s, annealing at 55 °C for 30 s, and extension at 72 °C for 5 s, followed by a final extension step at 72 °C for 5 min. Primers were designed based on the reference sequence of the bovine *ELF5* gene available in the NCBI database to amplify the genomic region containing the candidate polymorphic site. The sequences of primers for amplifying the *ELF5* g.32793 locus were: Forward 5′-TGGCTCAAGCTGTCAACACA-3′; Reverse 5′-TGCGTACCCATCAGGTAGGA-3′.

Following PCR amplification, the PCR products were purified and submitted to Sangon Biotech (Shanghai, China) for Sanger sequencing.

### 2.5. Kompetitive Allele-Specific PCR (KASP)

The KASP genotyping assay was carried out using a touchdown PCR program. PCRs contained 2× KASP Master Mix, two allele-specific forward primers (Primer 1 and Primer 2), a reverse primer, diluted genomic DNA template (20 ng), and ddH_2_O in a final volume of 10 μL. After amplification, fluorescence signals of FAM and HEX channels were collected at 30 °C using a real-time PCR system. Genotypes were automatically clustered and classified based on fluorescence intensity values: homozygous genotype TT for the FAM-labeled allele, homozygous genotype GG for the HEX-labeled allele, and heterozygous genotype GT carrying both alleles. Samples with ambiguous or low fluorescence signals were excluded from subsequent statistical analysis. The primer sequences used for KASP were as follows: Primer 1, Forward 5′-GAAGGTGACCAAGTTCATGCTGGTAGGAAAACAAAGGGAGAGAA-3′; Primer 2, Forward 5′-GAAGGTCGGAGTCAACGGATTGGTAGGAAAACAAAGGGAGAGAC-3′; Reverse 5′-CTGACGGCTCTGTAATAGGCAA. The quality of KASP genotyping was evaluated based on fluorescence clustering patterns. Clear separation of genotype clusters was observed, and all samples were successfully assigned to corresponding genotypes. In addition, representative samples with different genotypes were randomly selected for Sanger sequencing validation, and the sequencing results were consistent with the KASP genotyping results, confirming the accuracy of the genotyping assay.

### 2.6. MAC-T Cell Proliferation Assay Using EdU Assay

In this study, the EdU assay was performed to evaluate the proliferation ability of bovine mammary epithelial cells. The EdU detection in this study was conducted using the EdU Kit (Beyotime, C0075S, Shanghai, China). First, cells were exposed to pre-warmed EdU working solution for 2 h. After incubation, fixation was carried out using 4% paraformaldehyde for 15–20 min, then washed three times with PBS (Mishushengwu, MI00625, Shaanxi, China). And using 0.3% Triton X-100 (Solarbio, T8205, Beijing, China) was used for permeabilization for 20 min, and subsequently incubated with the EdU reaction solution and nuclear stain. Fluorescence microscopy was used for observation; quantitative analysis was carried out through Fiji Image J (V 1.54H).

### 2.7. Cell Cycle and Flow Analysis

Cell cycle distribution was evaluated using the Cell Cycle Detection Kit (MCE, HY-K1071, Monmouth Junction, NJ, USA) in accordance with the manufacturer’s protocol. Collected cells were added to 1 mL of ice-cold 70% ethanol, gently mixed, and fixed at 4 °C. Then centrifuging for 5 min to obtain the cell pellet. Using 1 mL of ice-cold PBS to wash cells again. After the supernatant was removed while retaining approximately 50 μL PBS, the tube was gently flicked to disperse the cells. Each sample was incubated with 0.5 mL PI solution, followed by gentle and thorough resuspension of the cell pellet. Samples were incubated for 30 min. Flow cytometric analysis was performed following PI staining. All data were processed and analyzed with the appropriate software integrated with the FongCyte™ flow cytometer (Challen Biotechnology, Beijing, China).

### 2.8. Western Blot

Cells were washed with PBS and lysed with RIPA lysis buffer (Solarbio, R0010, Beijing, China) containing a protease inhibitor cocktail. Approximately 1 × 10^6^ cells were lysed in 200 μL lysis buffer for 30 min on ice. The lysates were centrifuged at 12,000 rpm for 15 min at 4 °C to remove cell debris, and the supernatants were collected. Protein concentrations were determined using the BCA protein assay kit (Epizyme, ZJ101, Shanghai, China) according to the manufacturer’s instructions. Equal amounts of denatured protein (5× SDS loading buffer, 100 °C, 8 min) were separated by SDS-PAGE (85 V for stacking gel, 100 V for resolving gel) and transferred onto PVDF membranes (Millipore Corporation, IPVH00010, Billerica, MA, USA) through a wet transfer system (250 mA). The membranes were first blocked using Protein-Free Rapid Blocking Solution (Epizyme, PS108P, Shanghai, China)) for 15 min, followed by three washes with TBST (3 × 5 min). Subsequently, primary antibodies were incubated overnight at 4 °C, and the membranes were then treated with secondary antibodies for 2 h at room temperature. Protein bands were detected using a chemiluminescence system and visualized with GV90L-E Chemiluminescence Imaging System (BLT BioLight Technology, Guangzhou, China). The relative protein expression levels were quantified by Fiji Image J software with internal reference proteins for normalization.

The primary antibodies used in this study were as follows: anti-CDK2 antibody (Huabio, ET1602-6, 1:1000 dilution, Hangzhou, Zhejiang, China), anti-PCNA antibody (Huabio, ET1605-38, 1:5000 dilution, Hangzhou, Zhejiang, China), anti-CCND1 antibody (Wanlei, WL01435a, 1:500 dilution, Dalian, Liaoning, China), and anti-β-actin antibody (Proteintech, 66009-I-Ig, 1:100,000 dilution, Proteintech Group, Chicago, IL, USA).

### 2.9. Transfection

The miRNA mimics and inhibitors were synthesized, and the dual-luciferase reporter vectors were constructed by Sangon Biotech (Shanghai, China).

Cell transfection was performed when the cell confluence reached approximately 70%. Cells were transfected with miRNA mimics, inhibitors, or plasmid DNA using Lipofectamine 2000 (Thermo Fisher Scientific, 11668019, Waltham, MA, USA) according to the manufacturer’s instructions. Briefly, nucleic acids were diluted in Opti-MEM (Gibco, 51985091, Waltham, MA, USA) and mixed with Lipofectamine 2000 to form transfection complexes. After incubation, the complexes were added to cells cultured in serum-free and antibiotic-free medium. After 6 h of incubation, the medium was replaced with complete culture medium, and cells were maintained under standard culture conditions for subsequent experiments.

### 2.10. Dual-Luciferase Reporter Assays

Dual-luciferase reporter assays were performed using a dual-luciferase reporter assay kit (Yeasen, 11402ES60, Shanghai, China) according to the manufacturer’s instructions. Briefly, after transfection, cell lysates were collected, and the activities of firefly and Renilla luciferases were sequentially measured. The relative luciferase activity was calculated as the ratio of firefly luciferase activity to Renilla luciferase activity (F/R).

### 2.11. Data Analysis

The genetic diversity parameters of the identified polymorphic loci were analyzed based on allele frequencies according to the method described by Nei [15]. The observed heterozygosity (Ho), effective allele number (Ne), and polymorphism information content (PIC) were calculated to evaluate the genetic variation in the population.

Statistical analyses were conducted using SPSS software (version R26.0.0.0; IBM Corp., Armonk, NY, USA) and GraphPad Prism (version 10.1.2; GraphPad Software, San Diego, CA, USA). All cell-based experiments were performed with three independent biological replicates. Quantitative data from Western blot densitometric analysis and flow cytometric cell cycle analysis are presented as mean ± standard deviation (SD). Statistical significance was determined using appropriate statistical tests, with *p* < 0.05, *p* < 0.01, and *p* < 0.001 considered statistically significant.

For cellular and molecular experiments, differences between two groups were assessed using an unpaired two-tailed Student’s *t*-test, while comparisons involving more than two groups were performed using one-way analysis of variance (ANOVA).

The association between the *ELF5* g.32793 G>T polymorphism and lactation traits was evaluated using a general linear model (GLM). Genotype, parity, calving year, testing season, and lactation stage were included as fixed effects in the model. The statistical model was defined as Y_ijklmn_ = μ + G_i_ + P_j_ + CY_k_ + S_l_ + LS_m_ + e_ijklmn_, where Y_ijklmn_ represents the observed milk production traits; μ represents the overall mean; G_i_ represents the genotype effect; P_j_ represents the parity effect; CY_k_ represents the calving year effect; S_l_ represents the testing season effect; LS_m_ represents the lactation stage effect; and e_ijklmn_ represents the residual error. Type III sums of squares were applied to correct for unequal sample sizes among genotype groups. Because all animals were derived from the same herd and maintained under the same feeding and management conditions, herd and management effects were not included as independent fixed effects. Age was not included as an independent factor because it was highly correlated with parity. FDR correction was performed to control false positives caused by multiple trait testing. When significant differences among genotypes were detected, Duncan’s multiple range test was conducted for pairwise comparisons among genotype means. Statistical significance was defined as *p* < 0.05.

## 3. Results

### 3.1. Polymorphism Identification and Genetic Parameter Analysis at the g.32793 of ELF5

**Figure 1:**
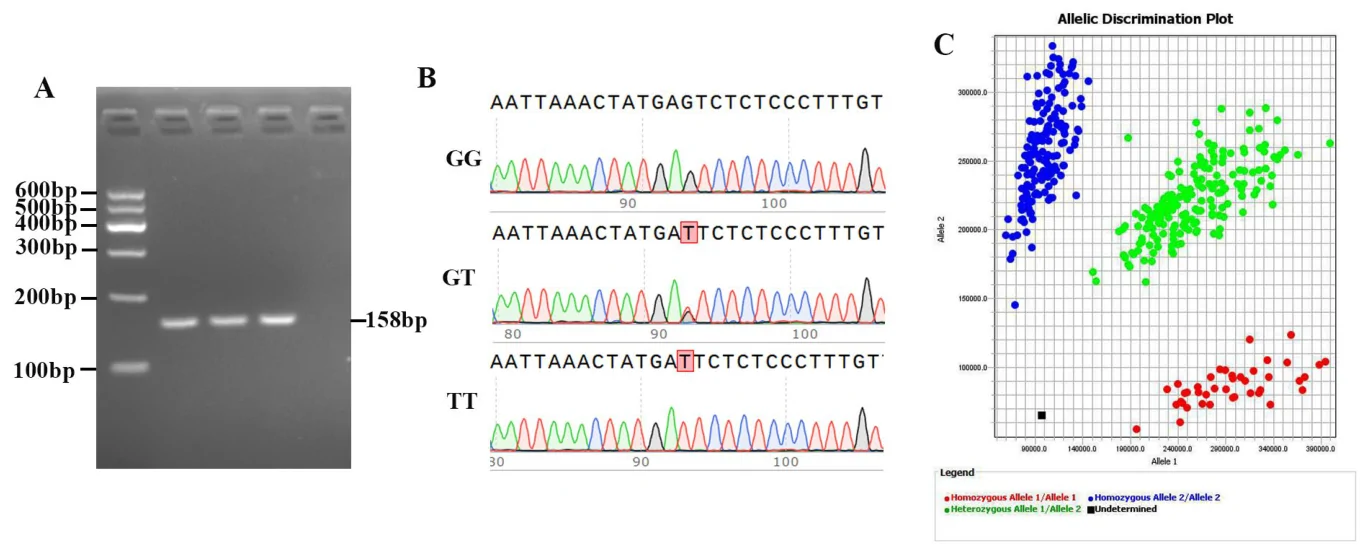
Polymorphism identification of *ELF5* g.32793. (**A**) The PCR product of *ELF5* g.32793 was resolved on a 3% agarose gel. (**B**) Sanger sequencing of the *ELF5* g.327939 (G>T) locus. (**C**) Genotyping of the locus was carried out using the KASP genotyping technique. The results represent three biological replicates (mean ± SD).

We amplified the PCR product of 158 bp to detect the mutation at the g.32793 site of *ELF5* via PCR and agarose gel electrophoresis (Figure 1A). Double peaks were observed at this site of *ELF5* by Sanger sequencing, indicating a clear base mutation (G>T) at this locus (Figure 1B). Next, the KASP genotyping technique was performed to genotype this locus. Based on the fluorescence clustering plot (Figure 1C), the red cluster indicates individuals homozygous for the TT allele, the blue cluster indicates GG homozygous individuals, and the green cluster indicates GT heterozygous individuals. Therefore, the experimental population could be classified into three genotypes: GG, GT, and TT.

The population genetic parameters of the *ELF5* g.32793 locus in Holstein cattle were analyzed, including the calculation of genotype and allele frequencies. At the *ELF5* g.32793 (G>T) locus, the G allele occurred at a markedly higher frequency than the T allele. The GG and GT genotypes were the most prevalent, with genotype frequencies of 0.414 and 0.479; the TT genotype, which was the least frequent, had a frequency of 0.107. Additionally, the observed heterozygosity (Ho), expected heterozygosity (He), effective allele number (Ne), and polymorphism information content (PIC) for this locus were 0.479, 0.453, 1.829, and 0.350, respectively. The g.32793 (G>T) locus in the *ELF5* gene showed moderate polymorphism (0.25 ≤ PIC ≤ 0.5). The locus deviated significantly from Hardy–Weinberg equilibrium (*p* < 0.05) (Table 1).

### 3.2. ELF5 g.32793 (G>T) Variant and Its Association with Milk Traits

The *ELF5* g.32793 (G>T) SNP is associated with milk traits, including daily MY, 305-day MY, MFP, MPP and SCS, as presented in Table 2. The GG genotype of this locus was significantly associated with daily MY, 305-day MY, MPP and SCS (*p* < 0.05). Multiple comparison analysis showed that individuals with the GT genotype had significantly higher daily MY and 305-day MY compared with those carrying GG and TT genotypes (*p* < 0.05). However, the MFP and MPP in individuals with the GT genotype were significantly decreased. Overall, the GT genotype at this locus appears to be a key genetic factor for improving milk yield, but its impact on milk composition highlights the complexity of breeding decisions, where optimizing daily MY may require balancing with milk quality traits. Furthermore, we found that individuals with the TT and GG genotypes exhibited higher MFP and MPP than those with the GT genotype, but lower daily MY and 305-day MY. Although significant differences were detected among genotypes, the relatively small phenotypic variation suggests that these variants may have limited individual effects on milk traits. Nevertheless, they may serve as potential genetic markers for further investigation of lactation-related traits.

### 3.3. The Mutation ELF5 g.32793 (G>T) Affects the Binding of bta-miR-487a to Its Target

**Figure 2:**
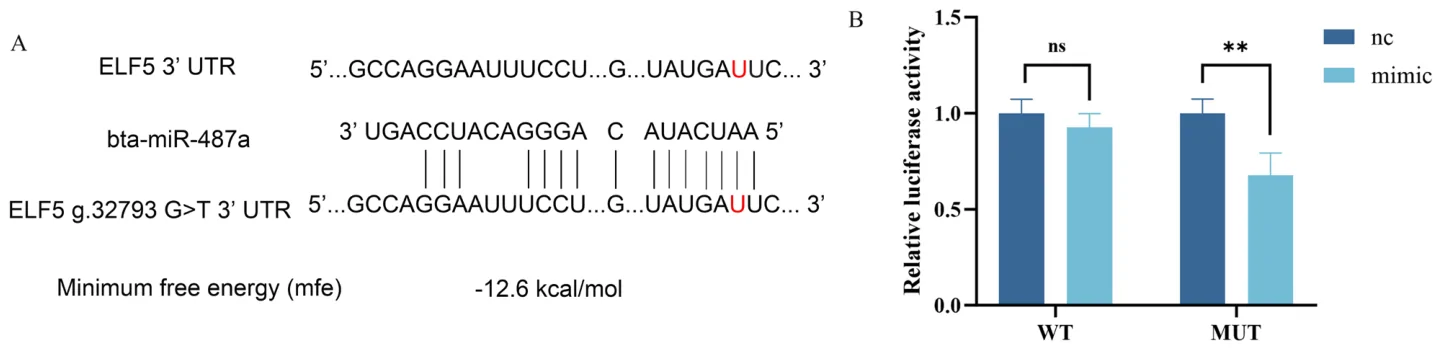
bta-miR-487a binds to the 3′ UTR region of *ELF5*. (**A**) Schematic diagram of bta-miR-487a binding to the 3′ UTR region of *ELF5*, the red letters indicate mutation sites. (**B**) Co-transfected with WT or MUT and mimic or nc in the HEK-293 T cell line, and the fluorescence activity was detected by the dual luciferase assay. The results represent three biological replicates (mean ± SD), ns = not significant, ** *p* < 0.01.

Bta-miR-487a was predicted to bind to the 3′ UTR region of *ELF5* by RNAhybrid; the information for it was obtained from the miRBase database (Figure 2A). The site affects the binding of bta-miR-487a to the 3′ UTR region of *ELF5* by constructing a dual luciferase reporter containing the 3′ UTR region of *ELF5*, which is wild type (WT), and a mutant vector (MUT) that mutates the site g.32793 G to T. The results showed that the mimic of bta-miR-487a did not affect the fluorescence activity of WT, but affected the fluorescence activity of MUT; thus, the *ELF5* g.32793 (G>T) variant enhanced the binding of bta-miR-487a to the *ELF5* 3′ UTR region (Figure 2B). Furthermore, the genotype of the MAC-T cells at this locus was identified as G by Sanger sequencing (Figure S1A). Combined with the above results, overexpression or inhibition of bta-miR-487a did not affect *ELF5* mRNA expression in MAC-T cells (Figure S1B,C), further demonstrating that this locus affects the interaction between bta-miR-487a and the *ELF5* 3′ UTR.

### 3.4. bta-miR-487a Inhibits Cell Proliferation of MAC-T Cells by Arresting the Cell Cycle in the G1 Phase

**Figure 3:**
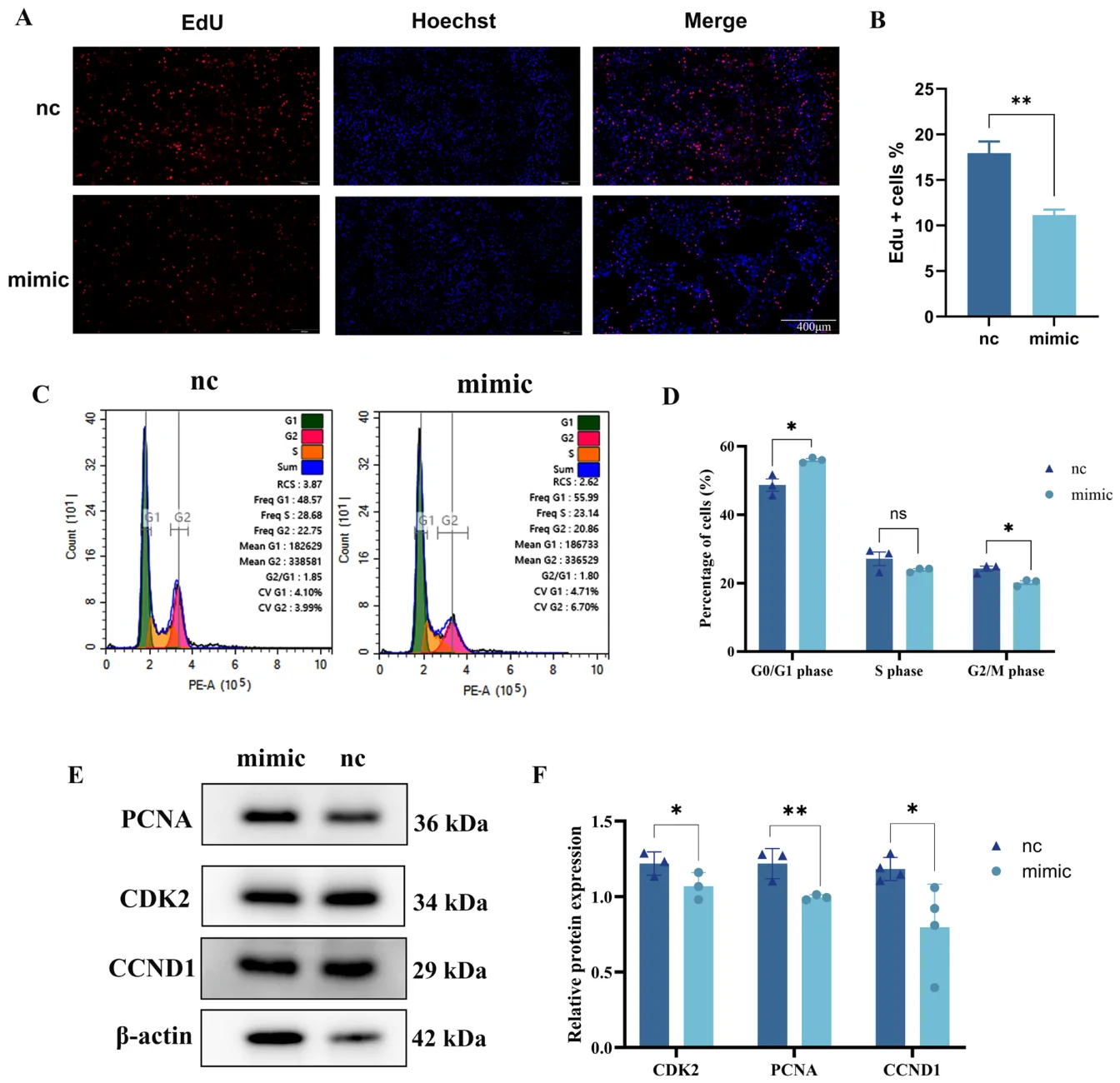
Overexpression of bta-miR-487a inhibits MAC-T cell proliferation. (**A**) EdU assay for the proliferation of MAC-T cells overexpressing bta-miR-487a, red indicates EdU-positive cells, and blue indicates nuclei. (**B**) Quantitative analysis of EdU-positive cells in the EdU assay. (**C**) Cell cycle distribution of MAC-T cells overexpressing bta-miR-487a was analyzed by flow cytometry. (**D**) Quantitative analysis of cell cycle distribution in flow cytometry. (**E**) Expression levels of proliferation-related proteins in MAC-T cells overexpressing bta-miR-487a. Full-length bovine CCND1 has a theoretical molecular weight of 33.7 kDa (standard nominal size 34 kDa). The antibody targets the central domain of CCND1, and the detected band displayed an apparent molecular weight of ~29 kDa. (**F**) Grayscale quantification of the Western blot. The results represent three biological replicates (mean ± SD), ns = not significant, * *p* < 0.05, ** *p* < 0.01. Original WB images can be found in Supplementary Materials.

To investigate the effect of bta-miR-487a on the proliferation of MAC-T cells, an EdU experiment was performed. Overexpression of bta-miR-487a significantly inhibited MAC-T cell proliferation (Figure 3A,B). Flow cytometry analysis further showed that bta-miR-487a overexpression significantly increased the proportion of cells in the G0/G1-phase while decreasing the proportion of G2/M-phase, indicating G0/G1 phase cell cycle arrest (Figure 3C,D). Consistently, Western blot analysis demonstrated that overexpression of bta-miR-487a significantly downregulated the protein expression levels of PCNA, CCND1, and CDK2 (Figure 3E,F). In addition, overexpression of bta-miR-487a significantly reduced the mRNA expression levels of CSN2 and CSN1S1 in MAC-T cells (Figure S1D,E).

**Figure 4:**
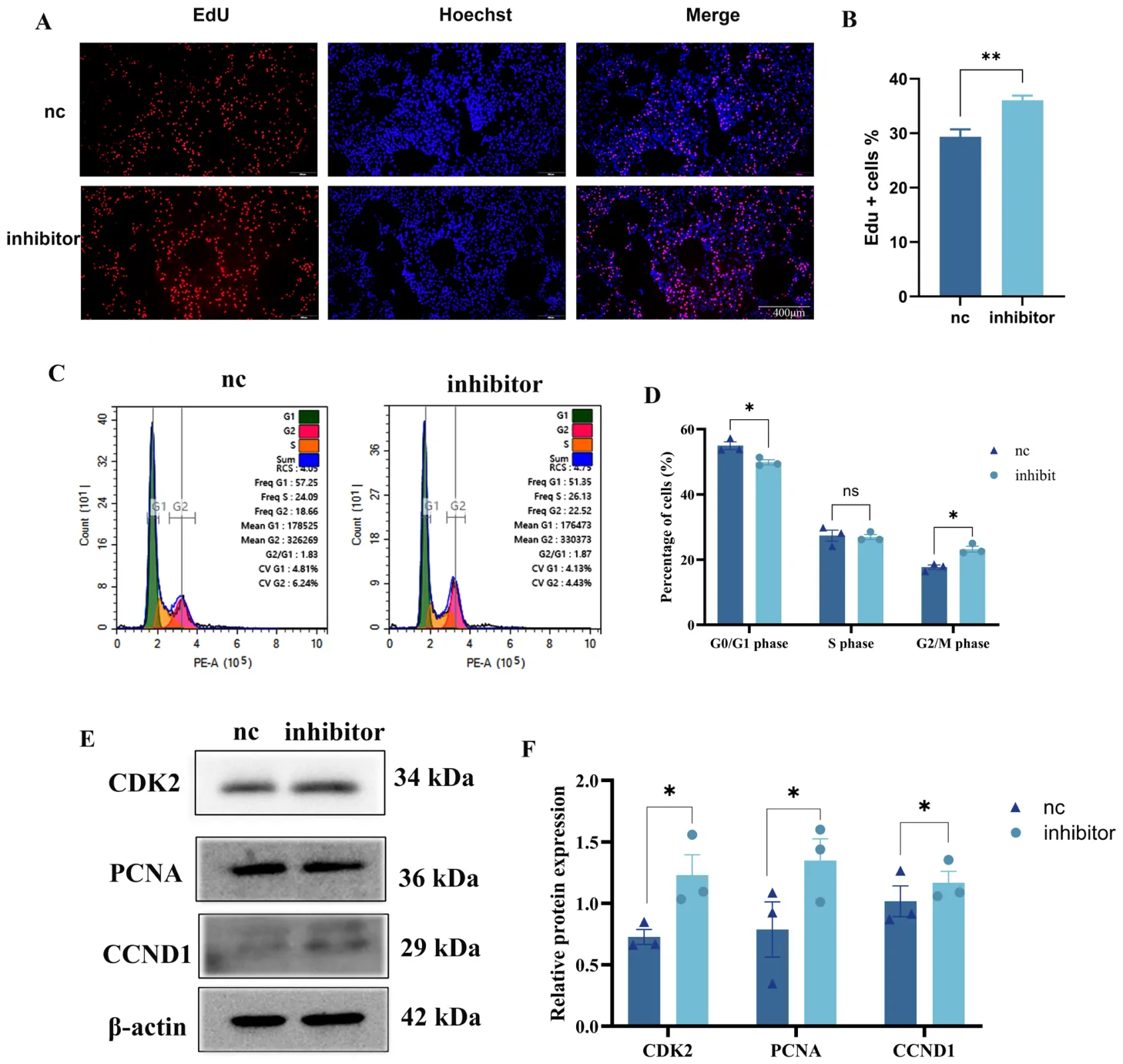
Knockdown of bta-miR-487a promotes MAC-T cell proliferation. (**A**) EdU assay for proliferation of MAC-T cells with knockdown of bta-miR-487a, red indicates EdU-positive cells, and blue indicates nuclei. (**B**) Quantitative analysis of EdU-positive cells in the EdU assay. (**C**) Cell cycle distribution of MAC-T cells with knockdown of bta-miR-487a was analyzed by flow cytometry. (**D**) Quantitative analysis of the cell cycle distribution by flow cytometry. (**E**) Expression levels of proliferation-related proteins in MAC-T cells with knockdown of bta-miR-487a. (**F**) Grayscale quantification of the Western blot. The results represent three biological replicates (mean ± SD), ns = not significant, * *p* < 0.05, ** *p* < 0.01. Original WB images can be found in Supplementary Materials.

In contrast, knockdown of bta-miR-487a significantly promoted MAC-T cell proliferation (Figure 4A,B). Flow cytometry resulted in the opposite cell cycle distribution, with a decreased proportion of G0/G1-phase cells and an increased proportion of G2/M-phase cells, indicating enhanced cell cycle progression after bta-miR-487a knockdown (Figure 4C,D). Also, knockdown of bta-miR-487a significantly increased protein expression levels of PCNA, CCND1, and CDK2 (Figure 4E,F). Moreover, suppression of bta-miR-487a significantly increased the mRNA expression levels of CSN2 and CSN1S1 (Figure S1D,E). Collectively, the results suggested that bta-miR-487a may play an important role in regulating mammary epithelial cell function.

## 4. Discussion

A novel SNP locus, *ELF5* g.32793 (G>T), was identified in this study and subsequently genotyped in 1314 Holstein dairy cattle. At the *ELF5* g.32793 (G>T) locus, the frequency of the G allele was higher than that of the T allele. The GG and GT genotypes were more prevalent than the TT genotype. The PIC of the variant was 0.325, indicating moderate polymorphism. On the other hand, the observed genotype frequencies at this locus significantly deviated from HWE (*p* < 0.05), which can be attributed to several factors. First, Holstein cattle have been intensively selected worldwide for high milk yield. To improve milk production, milk quality, disease resistance, and other economically important traits, the cattle often undergo a selection program for specific genotypes. Such selective pressure directly alters allele and genotype frequencies at targeted loci, resulting in deviations from HWE [16]. Second, in commercial dairy farms, artificial insemination and selective breeding strategies are commonly employed to optimize breeding goals rather than random mating. This mating scheme alters genotype frequencies, increasing homozygosity and decreasing heterozygosity (i.e., inbreeding), thereby causing deviations from HWE [17]. However, deviation from HWE may also result from other factors, including population stratification, relatedness among sampled individuals, sampling bias, and random genetic processes.

Previous studies have demonstrated that *ELF5* is specifically highly expressed in mammary alveolar epithelial cells and serves as a key molecular switch that determines the differentiation of mammary epithelial cells toward the secretory lineage [7]. During the onset of lactation, the expression level of *ELF5* is markedly increased, and *ELF5* functions as a direct transcriptional activator of key milk protein–encoding genes, such as β-casein (CSN2), αS1-casein (CSN1S1), and κ-casein (CSN3), through its specific binding to ETS consensus motifs located within the promoter regions of these milk protein genes [18]. Although previous studies have extensively characterized the biological functions of ELF5 in mammary epithelial differentiation and lactation regulation, the contribution of *ELF5* genetic variation to lactation-related traits remains largely unexplored in dairy cattle. Most previous investigations have focused on the transcriptional regulation and functional roles of *ELF5*, whereas whether naturally occurring variants within the *ELF5* locus affect its regulatory activity and phenotypic variation has not been fully elucidated. In the present study, association analysis between the *ELF5* g.32793 (G>T) locus and lactation traits revealed that this locus was significantly associated with daily MY, 305-day MY, MFP, MPP, and SCS. These findings are consistent with the previously reported biological functions of *ELF5*. Furthermore, we found that individuals carrying the GT genotype exhibited significantly higher daily MY and 305-day MY than those with the other two genotypes, suggesting that this genotype may be advantageous in practical dairy production. However, individuals with the GT genotype were also associated with lower MFP, MPP, and SCS. The negative relationship between MY and milk component percentages observed in this study may be partially explained by the dilution effect commonly observed in high-producing dairy cows, in which increased milk volume may reduce the concentrations of milk components [19]. However, this explanation was not directly evaluated in the present study, and further investigations are required to clarify the biological mechanisms underlying the balance between milk yield and milk composition traits, which can be considered a limitation of the current study. Collectively, these findings indicate that high MY is generally accompanied by reduced MF and MPP, consistent with previous studies suggesting that superior lactation performance may compromise nutrient allocation for milk components such as fat and protein.

Increasing evidence suggests that functional variants located in the 3′ UTRs can contribute to complex trait variation by modifying post-transcriptional regulation, particularly through altering miRNA binding sites. In dairy cattle, several functional 3′ UTR SNPs, including variants in OLR1 [20], AMPKγ1 [21], CSN3 [22], and LEP [23], have been reported to influence economically important traits by altering miRNA-mediated post-transcriptional regulation of target genes. However, whether *ELF5* 3′ UTR variants participate in lactation regulation through similar mechanisms remains unknown. Therefore, the identification of the *ELF5* g.32793 G>T variant in the present study provides new insights into the genetic regulation of lactation traits and suggests a potential role of miRNA-associated regulatory variation in dairy cattle.

In this study, we further identified that bta-miR-487a targets the *ELF5* g.32793 (G>T) locus, suggesting that this SNP may represent a functional regulatory variant. The nucleotide change could affect the binding affinity of bta-miR-487a to *ELF5*, thereby modulating *ELF5* expression and subsequently influencing related cellular processes. Functional assays demonstrated that bta-miR-487a regulates MAC-T cell proliferation by modulating the cell cycle, likely through inhibition of the G1/S transition, leading to suppressed DNA replication and cell proliferation. Corresponding changes in the expression of proliferation-related genes, including CDK2, CCND1, and PCNA, further support a role for bta-miR-487a in the regulation of mammary epithelial cell proliferation. Previous studies have shown that miR-487a primarily regulates the progression of osteosarcoma [24], and clear cell renal cell carcinoma [25] by targeting downstream genes, whereas its function in bovine mammary cells has not been reported, indicating cell type-specific roles for miR-487a.

The proliferative potential of mammary epithelial cells is tightly linked to lactation performance, since the total population of functional secretory epithelial cells directly influences and ultimately determines the overall capacity for milk synthesis [26]. Thus, based on the targeting relationship between bta-miR-487a and the *ELF5* g.32793 (G>T) variant, we proposed that this SNP may influence mammary epithelial cell proliferation via a miRNA-mediated post-transcriptional mechanism, thereby promoting differentiation toward a secretory lineage and ultimately affecting lactation traits. Our functional experiments showed that bta-miR-487a-mediated cell cycle regulation suppresses MAC-T cell proliferation, which may facilitate differentiation of mammary epithelial cells toward a secretory phenotype, enhancing MF and protein synthesis. Genotype analysis revealed that GT individuals exhibited higher daily MY and 305-day MY but relatively lower MPP and MFP, likely due to a dilution effect associated with high milk volume, whereas TT individuals showed moderate daily MY and 305-day MY but higher MPP and MFP, suggesting that limited proliferation under this genotype may favor enhanced secretory function [27]. These findings indicate that the *ELF5* g.32793 (G>T) locus may modulate the balance between proliferation and differentiation of mammary epithelial cells through miRNA-*ELF5* interactions, leading to a trade-off between milk yield and composition. This provides new molecular insights into the regulation of mammary epithelial cell proliferation and secretory function, and highlights a potential functional SNP target for genetic improvement in dairy cattle. In the current study, genotype-dependent *ELF5* expression was not evaluated, which can also be considered a limitation of the study. Future studies using genotype-defined mammary tissues or animal models, together with *ELF5* rescue experiments, are needed to further validate the regulatory mechanism proposed in this study.

## 5. Conclusions

In summary, we have investigated a functional SNP, *ELF5* g.32793 (G>T), which shows a significant association with lactation traits in Holstein dairy cattle. The T allele enhances the interaction between bta-miR-487a and the *ELF5* 3′ UTR, suggesting that this variant may contribute to the regulation of mammary epithelial cell proliferation through an miRNA-mediated mechanism. Through modulation of cell cycle progression and proliferation-related genes, bta-miR-487a may contribute to the balance between epithelial cell proliferation and functional differentiation, ultimately affecting milk yield and milk composition. These findings reveal a SNP-miRNA regulatory mechanism influencing lactation traits and provide a valuable candidate marker for molecular breeding in dairy cattle.

## Figures and Tables

**Table 1:** Genotype frequencies and genetic parameters of g.32793 (G>T) in the *ELF5* gene.

Locus	Genotype Frequencies			Allele Frequencies		Genetic Parameters				HWE
*ELF5* g.32793 (G>T)	GG	GT	TT	G	T	Ho	He	Ne	PIC	*p*-values
	0.414	0.479	0.107	0.653	0.347	0.479	0.453	1.829	0.350	<0.05
	(*n* = 544)	(*n* = 629)	(*n* = 141)							

Note: HWE, Hardy–Weinberg equilibrium.

**Table 2:** Relationship between the SNP of *ELF5* and milk production traits in Holstein cattle.

Locus	Lactation Traits	Genotype (Means ± SE)			
		GG (*n* = 544)	GT (*n* = 629)	TT (*n* = 141)	*p*-Value
*ELF5* g.32793 (G>T)	Daily MY (kg)	35.12 ^b^ ± 10.53	35.73 ^a^ ± 10.58	35.17 ^b^ ± 10.37	0.002
	305-day MY (kg)	10,543.26 ^a^ ± 1974.51	10,590.72 ^c^ ± 1982.43	10,427.64 ^b^ ± 1892.51	3.0 × 10^−5^
	MFP (%)	3.89 ^b^ ± 0.87	3.85 ^b^ ± 0.90	3.94 ^a^ ± 0.85	0.012
	MPP (%)	3.38 ^a^ ± 0.38	3.35 ^b^ ± 0.38	3.39 ^a^ ± 0.38	3.7 × 10^−5^
	SCS	2.55 ^b^ ± 1.92	2.48 ^b^ ± 1.91	2.68 ^a^ ± 2.03	2.6 × 10^−5^

Note: Values with different superscripts within the same row differ significantly at *p* < 0.05 (a, b, c), MY (milk yield), MFP (milk fat percentage), MPP (milk protein percentage), and SCS (somatic cell score).

## Data Availability

The datasets generated and/or analyzed during the current study are available from the corresponding author upon reasonable request.

## Supplementary Materials

The following supporting information can be downloaded at: https://www.mdpi.com/article/10.3390/biom16081163/s1, Figure S1: Bta-miR-487a is associated with the mRNA expression of CSN2 and CSN1S1. Original WB images can be found in Supplementary Materials.

## Abbreviations

The following abbreviations are used in this manuscript:

3′ UTR	3′ untranslated region
MY	milk yield
MFP	milk fat percentage
MPP	milk protein percentage
SCS	somatic cell score
*ELF5*	E74-like ETS transcription factor 5
miRNA	microRNA
PI	propidium iodide
SD	standard deviation
ANOVA	analysis of variance
KASP	Kompetitive Allele-Specific PCR
Ho	observed heterozygosity
He	expected heterozygosity
Ne	effective allele number
PIC	polymorphism information content
HWE	Hardy–Weinberg equilibrium
CSN2	β-casein
CSN1S1	αS1-casein
CSN3	κ-casein
