# Supplementary material for: A Functional SNP Variant of the 3′ UTR Within the ELF5 Gene Affects Lactation Traits Through bta-miR-487a-Mediated Regulation in Holstein Dairy Cattle
Source: Biomolecules. 2026 Aug 10;16(8):1163. doi: 10.3390/biom16081163 (PMC13511253; doi:10.3390/biom16081163)
Supplement: Supplementary file 1 [file biomolecules-16-01163-s001.zip › biomolecules-4412632-Figure S1.pdf]

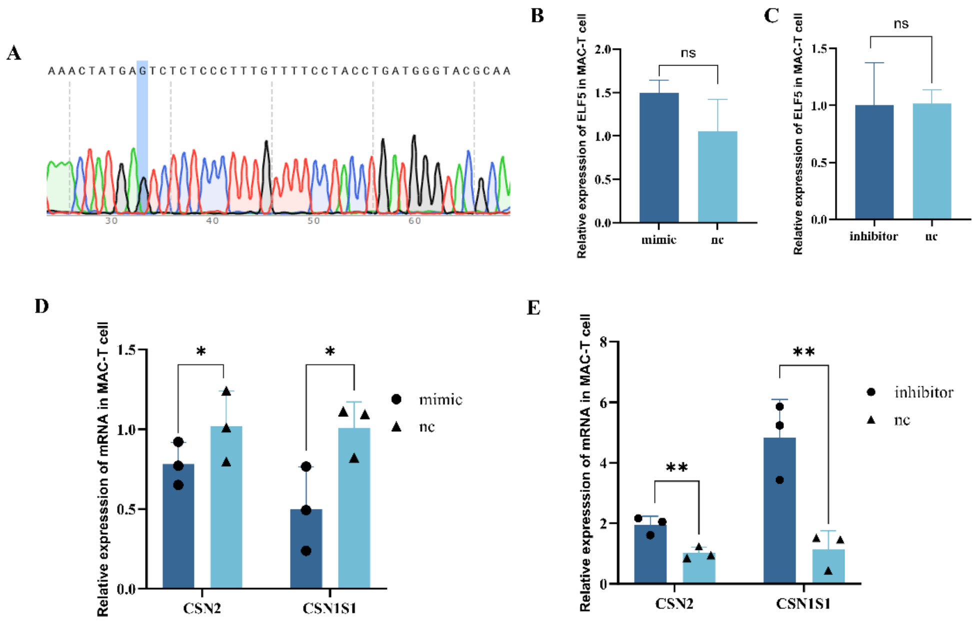

Figure S1. Bta-miR-487a is associated with the mRNA expression of CSN2 and CSN1S1. (A) The locus ELF5 g.32793 of MAC-T cell was G by sanger sequencing. (B-C) The mRNA expression level of ELF5 in MAC-T cell following bta-miR-487a overexpression or knockdown was detected by RT-qPCR. (D-E) The mRNA expression level of CSN1 and CSN1S1 in MAC-T cell following bta-miR-487a overexpression or knockdown were detected by RT-qPCR. The results represent three biological replicates (mean ± SD), **P* < 0.05, ***P* < 0.01, ****P* < 0.001.
