# Supplementary material for: A Functional SNP Variant of the 3′ UTR Within the ELF5 Gene Affects Lactation Traits Through bta-miR-487a-Mediated Regulation in Holstein Dairy Cattle
Source: Biomolecules. 2026 Aug 10;16(8):1163. doi: 10.3390/biom16081163 (PMC13511253; doi:10.3390/biom16081163)
Supplement: Supplementary file 1 [file biomolecules-16-01163-s001.zip › Original WB images/Fig.1A-Gel.pdf]

Fig. 1A

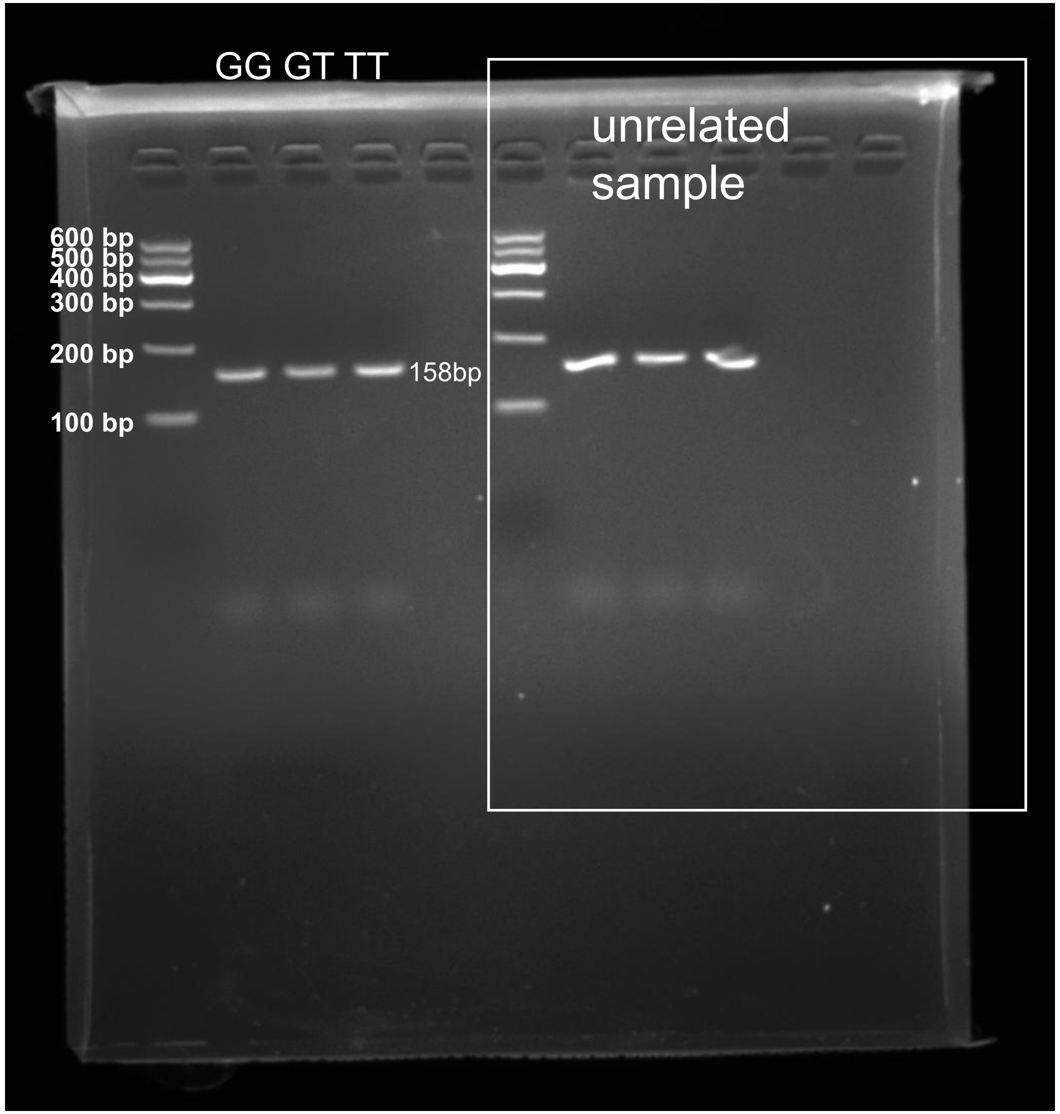
GG GT TT
unrelated sample
600 bp
500 bp
400 bp
300 bp
200 bp
100 bp
158bp
