# Supplementary material for: A Functional SNP Variant of the 3′ UTR Within the ELF5 Gene Affects Lactation Traits Through bta-miR-487a-Mediated Regulation in Holstein Dairy Cattle
Source: Biomolecules. 2026 Aug 10;16(8):1163. doi: 10.3390/biom16081163 (PMC13511253; doi:10.3390/biom16081163)
Supplement: Supplementary file 1 [file biomolecules-16-01163-s001.zip › Original WB images/Fig.3E and 4E-WB.pdf]

Fig.3E

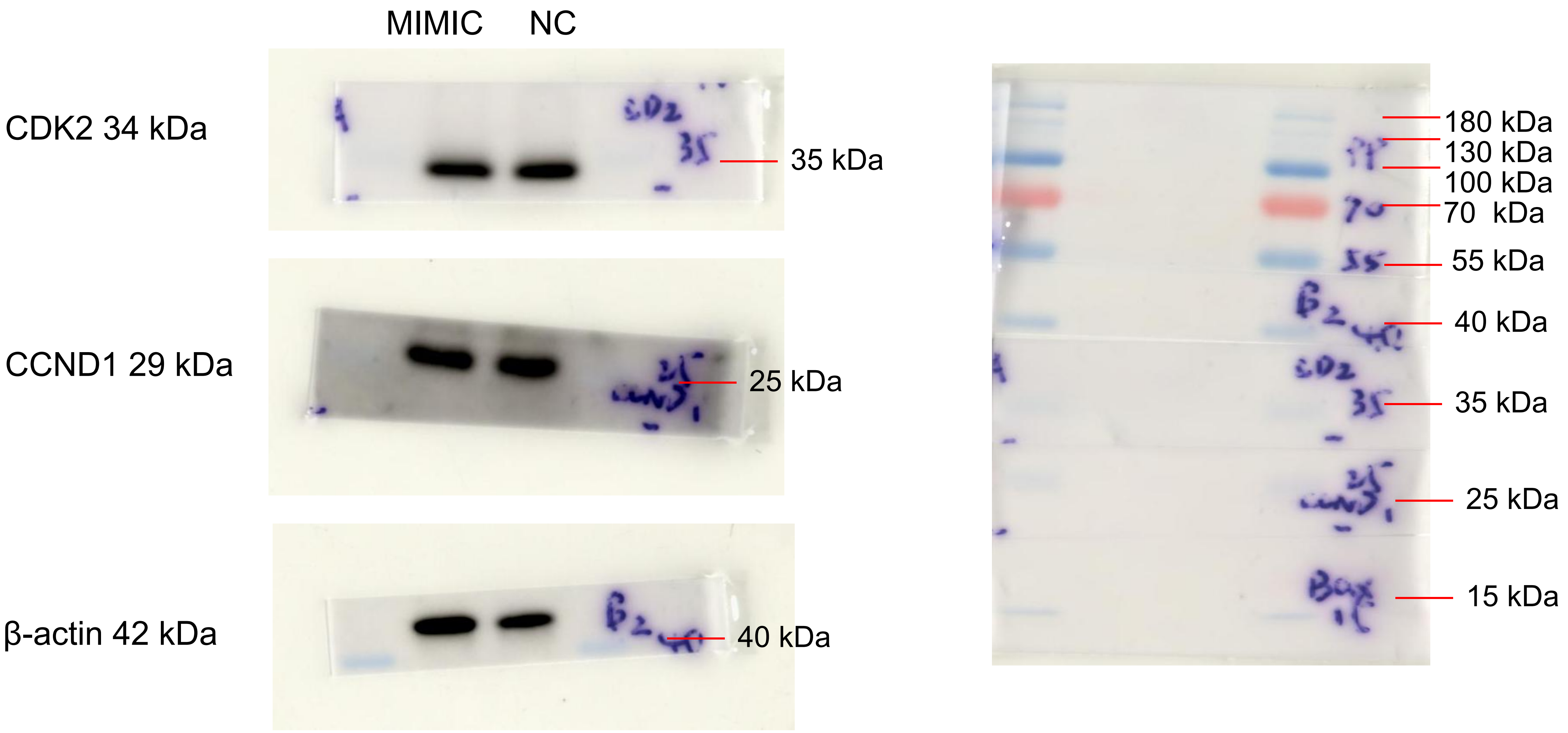

MIMIC
NC
CDK2 34 kDa
35 kDa
CCND1 29 kDa
25 kDa
β-actin 42 kDa
40 kDa
180 kDa
130 kDa
100 kDa
70 kDa
55 kDa
40 kDa
35 kDa
25 kDa
15 kDa

Fig.3E

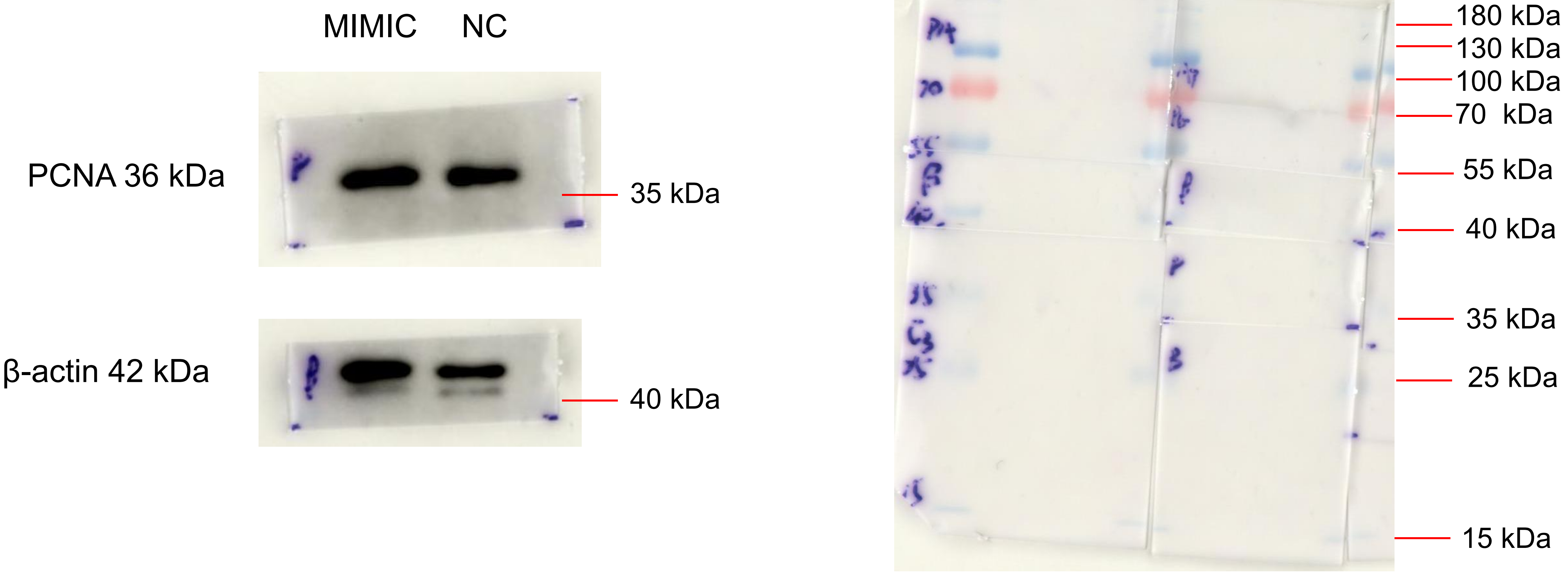

MIMIC
NC
PCNA 36 kDa
35 kDa
β-actin 42 kDa
40 kDa
180 kDa
130 kDa
100 kDa
70 kDa
55 kDa
40 kDa
35 kDa
25 kDa
15 kDa

Fig.4E

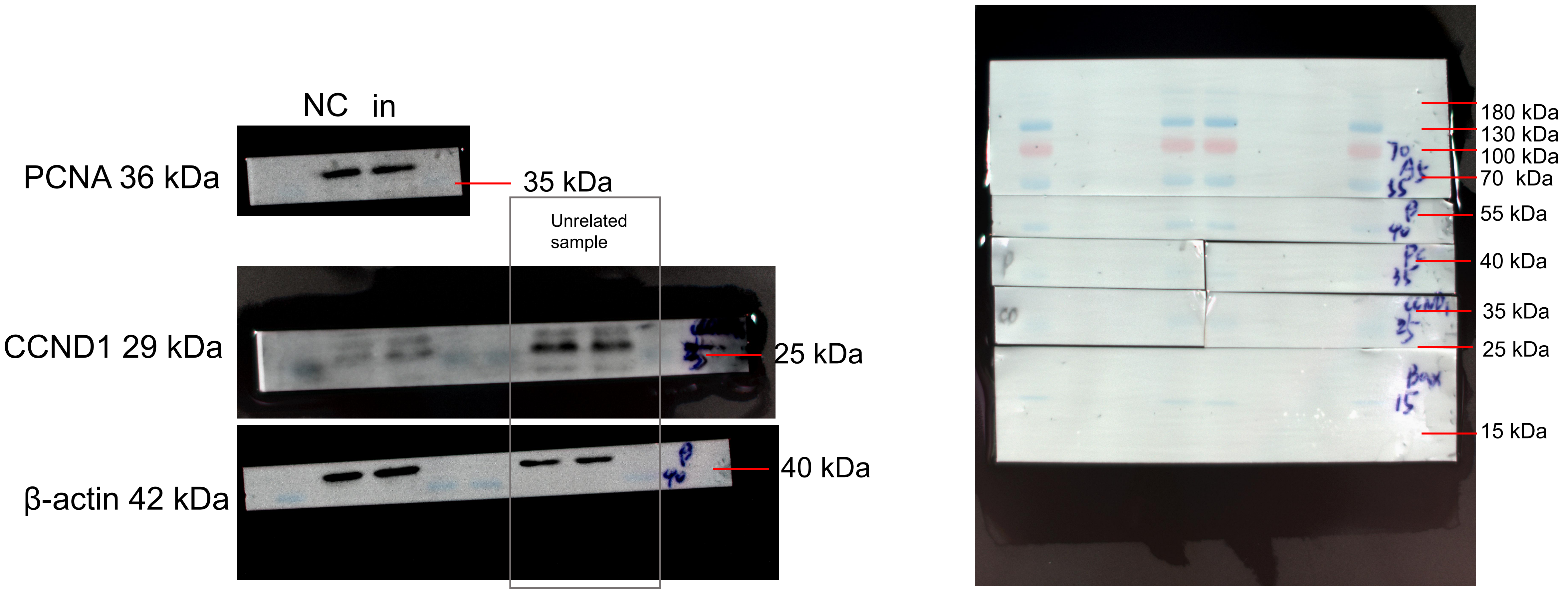
NC
in
PCNA 36 kDa
35 kDa
Unrelated sample
CCND1 29 kDa
25 kDa
β-actin 42 kDa
40 kDa
180 kDa
130 kDa
100 kDa
70 kDa
55 kDa
40 kDa
35 kDa
25 kDa
15 kDa

Fig.4E

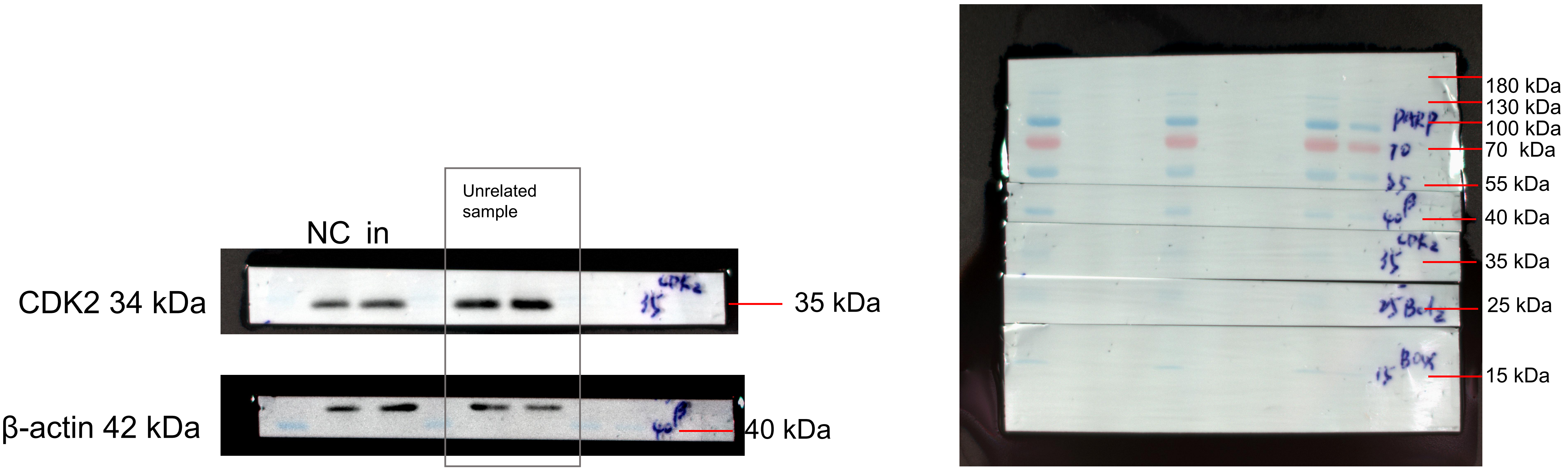
NC in
Unrelated sample
CDK2 34 kDa
35 kDa
β-actin 42 kDa
40 kDa
180 kDa
130 kDa
100 kDa
70 kDa
55 kDa
40 kDa
35 kDa
25 kDa
15 kDa
